# Full-thickness scalp and skull defect with dura mater exposure due to dissociation of pain sensation and anankastic personality disorder: a case report

**DOI:** 10.1080/23320885.2023.2285058

**Published:** 2023-11-28

**Authors:** Martynas Tamulevicius, Peter M. Vogt, Vincent Maerz

**Affiliations:** Department of Plastic, Aesthetic, Hand and Reconstructive Surgery, Hannover Medical School, Hannover, Germany

**Keywords:** Scalp defect, skull defect, chronic self-mutilation, anankastic personality disorder

## Abstract

Our case demonstrates a rare genesis of complex scalp defect with exposed dura mater in the occipital region due to self-mutilation. An early interdisciplinary approach is vital to prevent secondary complications and potentially fatal outcomes, particularly in psychiatric patients with reduced health awareness.

## Introduction

Full-thickness scalp and skull defects in the occipital region have been documented in the literature in the context of genetic syndromes with developmental disorders, destructive oncological processes, severe infections with accompanying destructive osteomyelitis, or past traumas [1, 2]. Self-mutilation as a cause of full-thickness defects has only been reported in patients with advanced dementia [3]. The dissociation of pain sensation in the affected area may contribute to potential self-mutilation. Additionally, systemic conditions such as diabetes mellitus, leading to diabetic polyneuropathy, can significantly reduce pain sensation or even trigger pruritus [4, 5].

Results from population-based psychiatric research have demonstrated a correlation between personality disorders and comorbidities such as chronic pain, sleep disorders, arthritis, arterial hypertension, as well as other cardiovascular and gastrointestinal diseases. With an incidence of 3–9%, obsessive-compulsive disorders, including anankastic personality disorder (APD), affect a significant portion of the population with psychiatric illnesses. Individuals suffering from these disorders experience unwanted, prolonged, overwhelming, and compulsive thoughts and/or behaviours. However, there is currently no definitive scientific clarification regarding the connection between psychiatrically diagnosed personality disorders and somatic systemic diseases [6–8].

Here, we present the first clinical case of a full-thickness scalp and skull defect with dura mater exposure resulting from self-mutilation in a patient with APD and dissociation of pain sensation, which developed over a period of 12 years.

## Case report

### Case history

**Figure 1.**
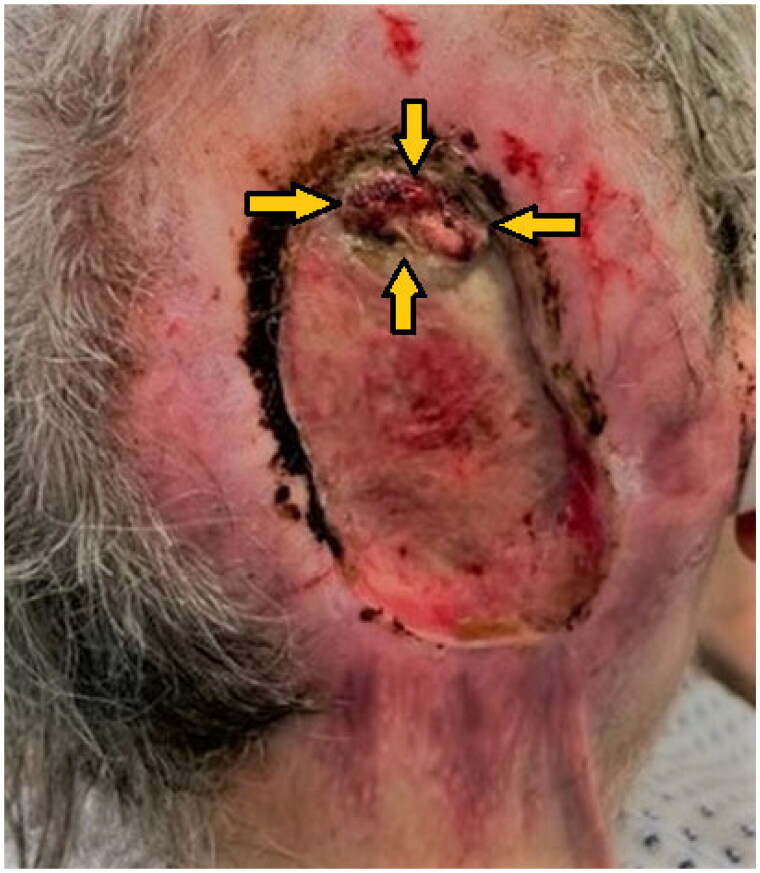
Full-thickness occipital skull defect with circular scar contractures and central exposure of the dura mater (indicated by arrows).

A 66-year-old patient was presented as an emergency case at an external neurological clinic due to noticeable neurological symptoms, including right-sided neglect. The patient had been unconsciously attempting to constantly turn to the right side while in bed for several hours. Additionally, a large oval skull cap defect with dura mater exposure in the occipital region was observed (see Figure 1). After ruling out intracranial bleeding, infarction, and cerebrospinal fluid circulation disorders through imaging, the patient was transferred to our clinic for evaluation of possible defect coverage.

According to the patient’s medical history, in 2010, he had been treated by otolaryngologists for an extensive nuchal abscess (approximately 10 × 10 cm) with early necrotizing fasciitis and bacteraemia, with positive blood cultures indicating Staphylococcus aureus (penicillin G-sensitive). After multiple debridements and partial wound closure, the clean and gut granulated wound was left to heal by secondary intention. Furthermore, the initial diagnosis of type II diabetes mellitus was made with significant hyperglycaemia (blood glucose level of 30.4 mmol/L, HbA1c of 10.8%). The antidiabetic therapy prescribed at that time was also discontinued by the patient himself 5 years ago, prior to the current presentation. Information obtained from third-party sources (general practitioner, family members, and close friends of the patient) revealed that the patient would frequently scratch his head with his fingers several times a day, often resulting in subungual bloodstains on his fingers. The patient reported experiencing persistent pruritus, especially in the occipital region, since the beginning of the wound healing by secondary intention in the nuchal region.

### Diagnostic and treatment

**Figure 2.**
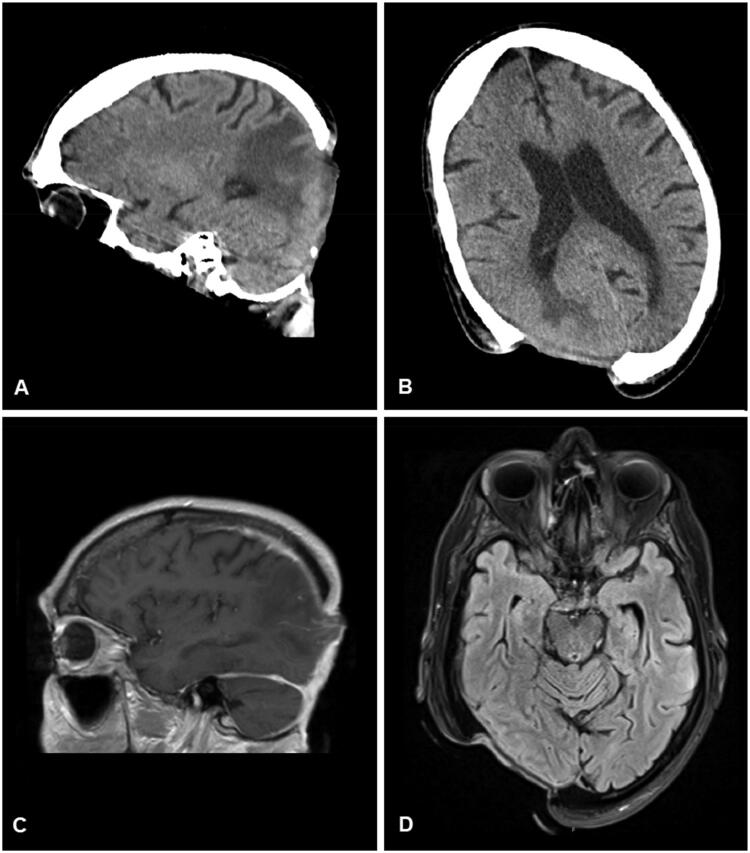
Computed tomographic (A and B) and magnetic resonance imaging (C and D) visualization of the skull defect.

**Figure 3.**
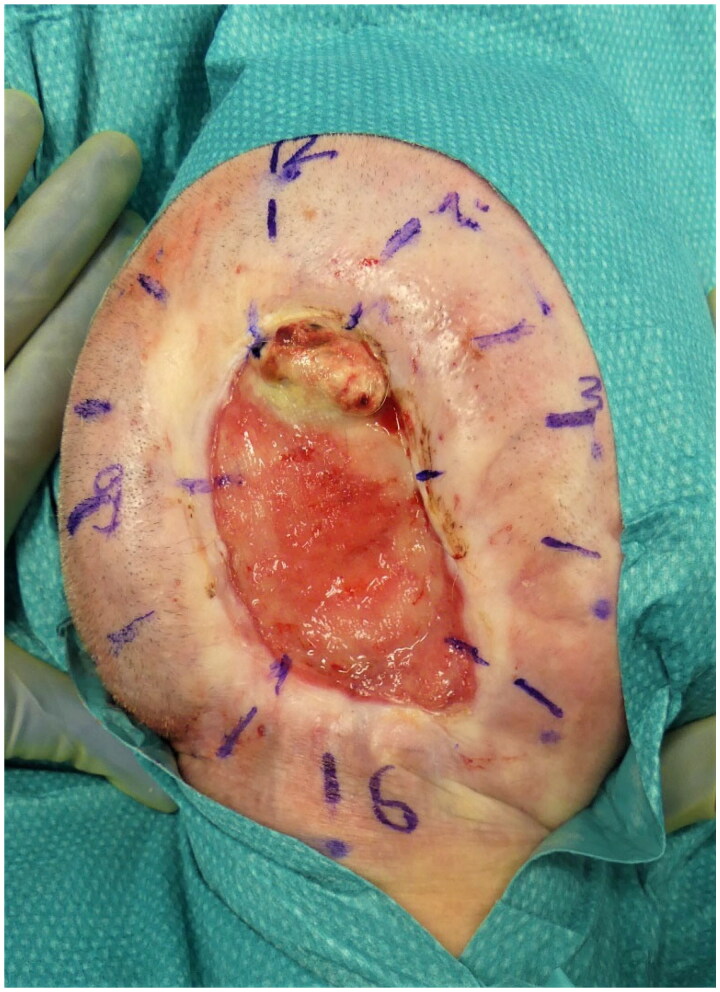
Field biopsy with the extraction of 12 cutaneous tissue samples from the wound margin.

The clinical examination revealed an oval, non-inflammatory scarred area (8.5 × 10 cm) in the high occipital region, painless upon palpation, with beginning scar retractions extending towards the nuchal area and central exposure of the dura mater (see Figure 1). There were no wounds observed in the nuchal region. Magnetic resonance imaging (MRI) and computed tomography (CT) scans were performed to rule out intracranial processes. Morphologically, a 5 × 7 cm scalp defect with chronically thickened dura mater was observed (see Figure 2). To exclude the possibility of scar carcinoma, a field biopsy was initially conducted, involving the collection of 12 cutaneous tissue samples from the wound margins (see Figure 3). Histopathological examination revealed a chronic inflammatory reaction of the dermis with acanthosis and hyperkeratosis, without evidence of malignancy.

Additionally, an interdisciplinary assessment of the poorly managed type II diabetes mellitus and psychiatric evaluation was conducted. With an HbA1c level of 7.2%, fasting glucose level of 6.8 mmol/L, and a long-standing diagnosis of diabetic polyneuropathy, metformin therapy was initiated. The psychiatric consultation confirmed APD. Furthermore, indications of the patient’s reduced health awareness and limited social engagement were observed.

**Figure 4.**
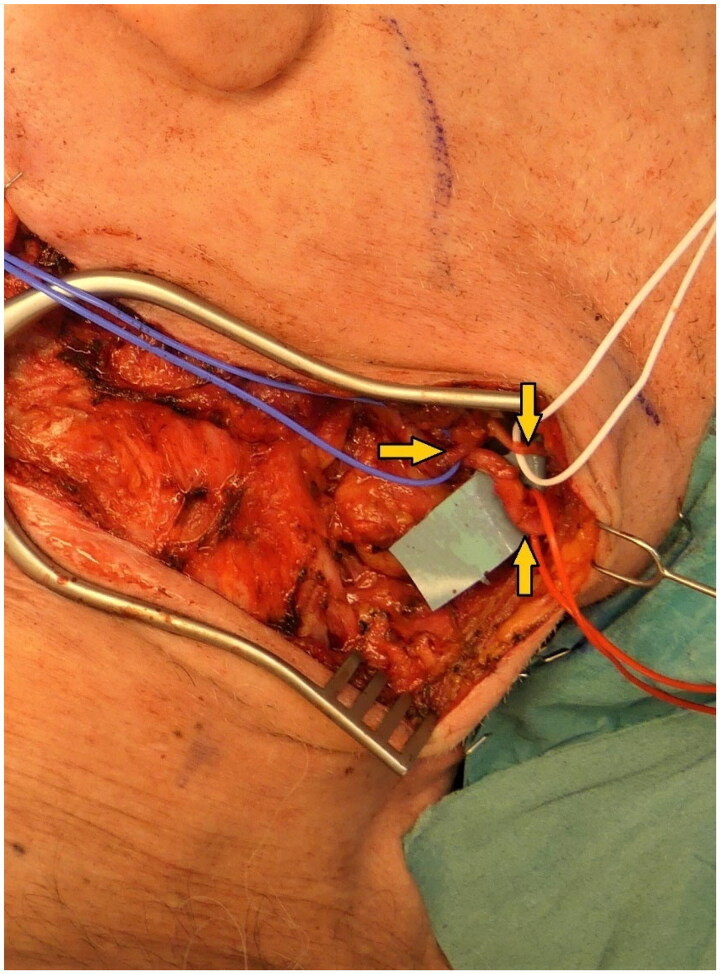
Defect coverage using a free myocutaneous latissimus dorsi flap with anastomosis to the right facial artery (intraoperatively). marked with arrows: facial artery and vein (red and blue rubber loops) and marginal mandibular branch of the facial nerve (white rubber loop).

**Figure 5.**
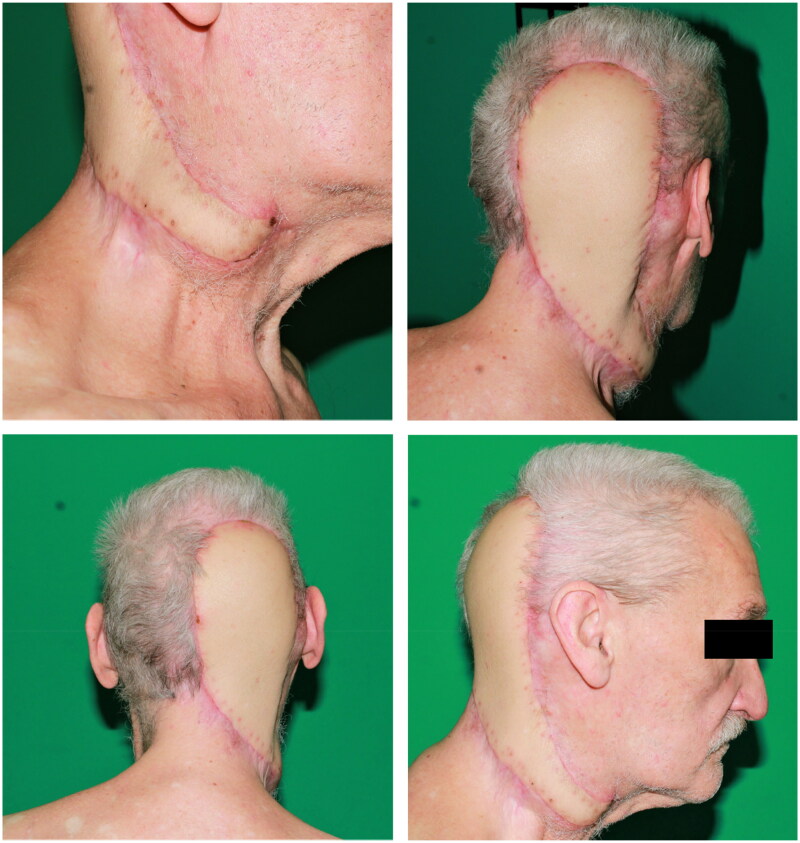
Postoperative result five weeks after defect coverage with a free myocutaneous latissimus dorsi flap.

After ruling out malignant processes, the all-layer defect in the occipital region was surgically managed through interdisciplinary collaboration. Following initial full-thickness debridement, the neurosurgery team performed a complication-free cranioplasty using bone cement (Palacos®; Heraeus Medical GmbH, Germany), and the existing skin-soft tissue defect (23 × 13 cm) was covered by plastic surgeons using a free myocutaneous latissimus dorsi flap, anastomosed to the right facial artery (see Figure 4). To maintain a postoperative medically-induced mean arterial blood pressure of 80 mmHg, the patient was closely monitored in the intensive care unit for several days. Postoperatively, a temporary neurapraxia of the marginal mandibular branch of the facial nerve was observed, resulting in right-sided drooping of the mouth corner, which regressed during follow-up examinations. The neurapraxia of the marginal mandibular branch of the facial nerve was considered iatrogenic, occurring during vascular preparation. The flap healed without signs of infection or other complications. Recurrent seroma aspirations were performed at the site of the latissimus dorsi flap donor site, which subsequently healed also without complications. Peri- and postoperatively, a calculated antibiotic therapy with amoxicillin and clavulanic acid was administered due to wound contamination with a sensitive Staphylococcus aureus strain. Outpatient diabetic and psychiatric follow-up care was arranged. At the 5-week postoperative follow-up examination, a well-perfused flap with non-irritated wound and scar conditions was observed (see Figure 5). Unfortunately, the patient did not show up for the scheduled follow-up appointments, so a comprehensive long-term follow-up was not possible.

## Discussion

A cranial defect with exposed dura mater caused by psychiatric-induced self-mutilation over a period of 12 years represents an extremely rare aetiology, according to the current literature. In cases of chronic wound defects, it is crucial to exclude the possibility of a tumour entity. Aplasia cutis congenita, despite its rarity, also presents significant etiological factor for cranial anomalies of this type. However, due to its predominantly antenatal or neonatal manifestation, this etiological origin was unlikely in this case. Malignant processes, such as basal cell carcinoma, squamous cell carcinoma (including scar carcinoma), or possible tumour metastasis, can underlie a variety of full-thickness bone-destructive processes in the cranial area [1].

So far, there are two case reports describing cranial defects resulting from uncontrolled scratching in the context of dementia syndrome and severe scalp pruritus due to post-herapeutic neuralgia [3, 9]. In both cases, the self-mutilation was attributed to an existing condition. In our case, the self-mutilation was accompanied by a dissociation of pain perception, presumably increased pruritus, and an undiagnosed APD.

Altered sensory perception of the scalp, especially regarding pain, can occur due to various conditions. These include nerve damage, neurological disorders, and skin diseases. There is limited data available on the frequency of scalp sensory disturbances [4, 5]. Some of the most common causes of reduced skin sensation include [4, 5]:

1. Traumatic head injuries with direct damage to the sensory nerves.
2. Systemic neuropathies (e.g., diabetic neuropathy).
3. Dermatoses such as herpes zoster and dermatitis.
4. Neurological disorders, such as multiple sclerosis and stroke.

In the described case, the patient also had untreated type II diabetes mellitus and a pre-existing diabetic polyneuropathy, which could be considered as a cause of altered sensitivity [5, 10]. Additionally, the patient reported chronic pruritus in the occipital region, which could be attributed to chronic irritation or damage of the left occipital minor and major nerves by surgical treatment of infection and during the secondary healing phase [10].

No direct association between obsessive-compulsive disorder (OCD) or APD and cranial defects has been described in the literature. Psychogenic dysesthesia is often described in the context of depression, anxiety, or other psychiatric disorders. It refers to pain dysesthesia that is elicited by psychological or emotional stimuli without a somatic stimulus, with pain qualities described as chronic, burning, tingling, or numbness [5]. Within the context of APD, compulsive behaviours can arise, leading to injuries or trauma to the head and skull. Self-mutilation, such as scratching or trichotillomania, can result in hair loss, scar formation, and chronic dermatoses. Avoidance behaviour is typical for individuals with APD. They try to avoid situations that are uncertain or unpredictable, including awareness of their own illness. Moreover, some patients with APD may refuse to seek help because they fear being perceived as weak or flawed or because they believe they should be able to cope with their symptoms on their own [8].

In summary, there are numerous conditions that can lead to self-inflicted wounds, making the success of surgical treatment a challenge in achieving long lasting closure due to their proneness to recurrence. Unfortunately, the follow-up duration for our patient was limited. Ideally, a follow-up period of at least 6 months or more is recommended for a comprehensive assessment. In cases where an extended follow-up is not feasible, during inpatient treatment, all potential conditions or risk factors should be evaluated, and preventive measures should be initiated before discharge.

## Conclusions

The case presented by us demonstrates a very rare genesis of a chronic wound healing disorder in the occipital region, caused by self-mutilation. The causes of such extensive defects are usually destructive oncological processes or severe traumas. In summary, chronic wounds, especially in patients with psychiatric disorders, should be critically and closely evaluated. A comprehensive assessment to exclude malignant conditions and early interdisciplinary reconstruction of complex cranial defects are recommended to reduce morbidity and should be preferred to secondary healing, especially in patients with reduced health awareness or other psychological limitations, to prevent secondary problems. Otherwise, a fatal outcome is possible.
